# Intramuscular desmoplastic fibroblastoma: a case report of extreme rarities in extremities

**DOI:** 10.1093/jscr/rjab461

**Published:** 2021-10-28

**Authors:** Louis Onghena, Lucas Verniers, Sofie Woussen, Caroline Van den Broecke, Kjell Fierens, Olivier Van Kerschaver

**Affiliations:** Department of General and Oncological Surgery, AZ Sint-Lucas Gent, Gent, Belgium; Department of Pathology, AZ Sint-Lucas Gent, Gent, Belgium; Department of Pathology, AZ Sint-Lucas Gent, Gent, Belgium; Department of Pathology, AZ Sint-Lucas Gent, Gent, Belgium; Department of General and Oncological Surgery, AZ Sint-Lucas Gent, Gent, Belgium; Department of General and Oncological Surgery, AZ Sint-Lucas Gent, Gent, Belgium

## Abstract

Soft tissue tumors are part of a wide and sometimes rare differential diagnostic landscape. Case description of these rare soft tissue masses helps the future differentiation and aids in preoperative multidisciplinary approach. Interpretation and staging, with the help of imaging, is key.

## INTRODUCTION

Desmoplastic fibroblastoma, also known as collagenous fibroma [1], is a rare benign soft tissue tumor [2, 3]. First described in 1995 [4], the mass is slow-growing without arguments for invasive local growth [5]. Desmoplastic fibroblastoma is a mass with a round or ovoid contour and lobulated texture with white to gray-scale surface [4, 6] and has been described in the following locations: subcutaneous, parenchymatous to intra-osseous, with muscular involvement in 27–45% [2, 4, 7]. The broad differential diagnosis includes other benign or low-grade fibroblastic or myofibroblastic lesions, post-traumatic myositis ossificans or fibrosis, low-grade fibromyxoid sarcoma and other benign fibromatous tumors [1].

## CASE DESCRIPTION

We describe the case of a 53-year-old woman with a mass in the right upper arm. Over the course of the last year, our patient noticed a slow-growing nodule. No pain or nervous symptoms were noted. On clinical examination, the mass is hard and positioned on/in the right-sided deltoid muscle. We suspect the mass to be slightly embedded in muscular tissue. Clinical examination is painless. No restriction in motion or alterations in sensibility are withheld. Our patient presents without relevant medical history and is an active, working woman.

### Imaging

**Figure 1:**
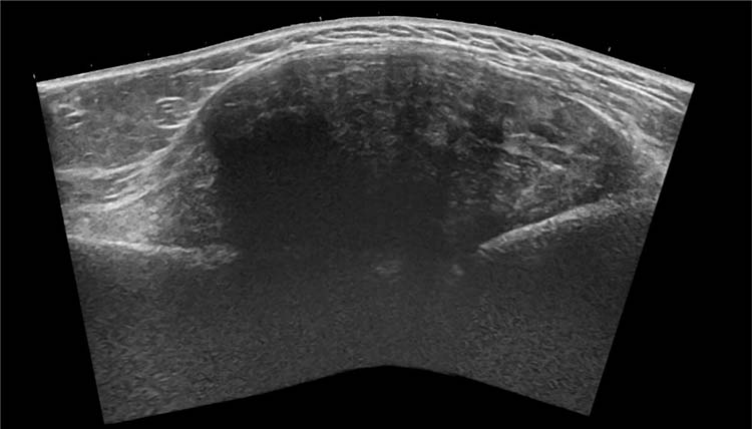
Sagittal panoramic US image of the right shoulder.

The general practitioner ordered an ultrasound (US) of the upper arm. US showed a sharp delineated fusiform mass (CC 8.5 × LL 6.5 × AP 3 cm) with heterogeneous echogenicity, associated with the lateral humeral cortex beneath the deltoid muscle (Fig. 1). The mass showed limited internal Doppler signal. A soft tissue tumor such as a sarcoma or desmoid was suspected, and magnetic resonance imaging (MRI) was advised.

**Figure 2:**
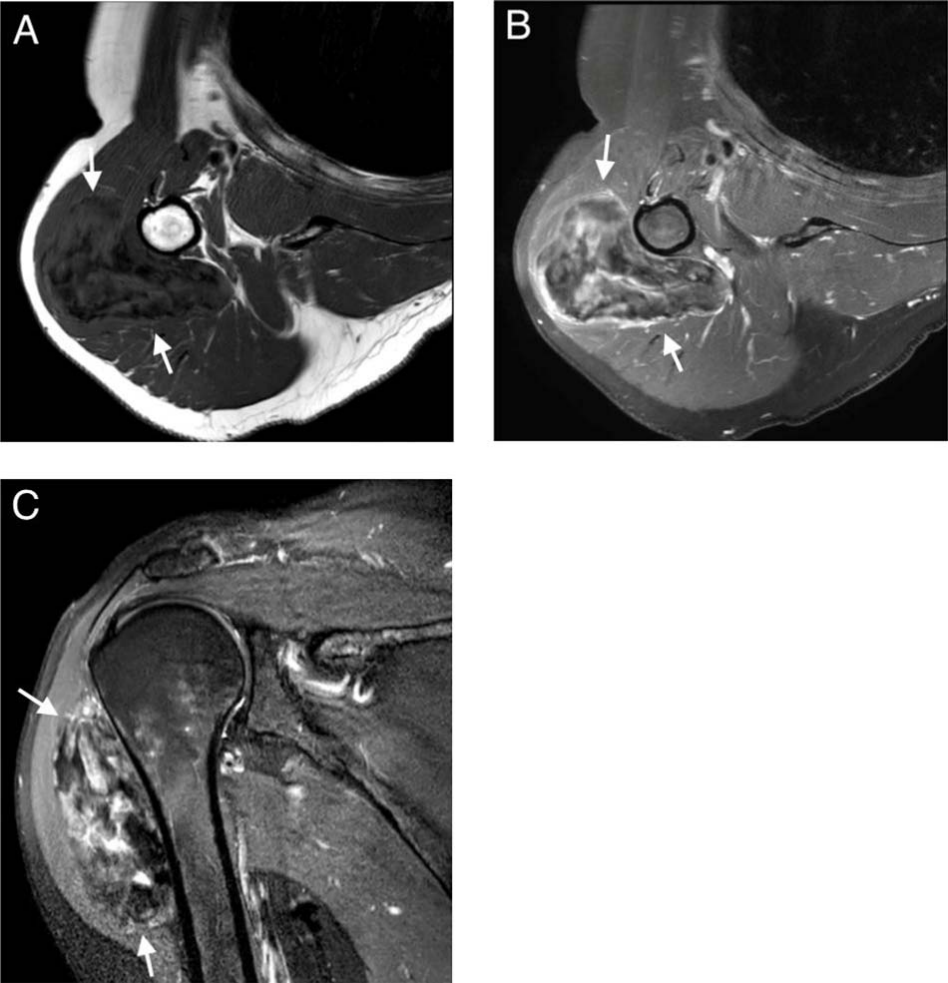
MRI with (**A**); axial T1-WI; (**B**) axial T1-FS-WI after administration of intravenous gadolinium contrast and (**C**) sagittal T2-FS-WI. Heterogeneous tumoral mass indicated with long arrows.

**Figure 3:**
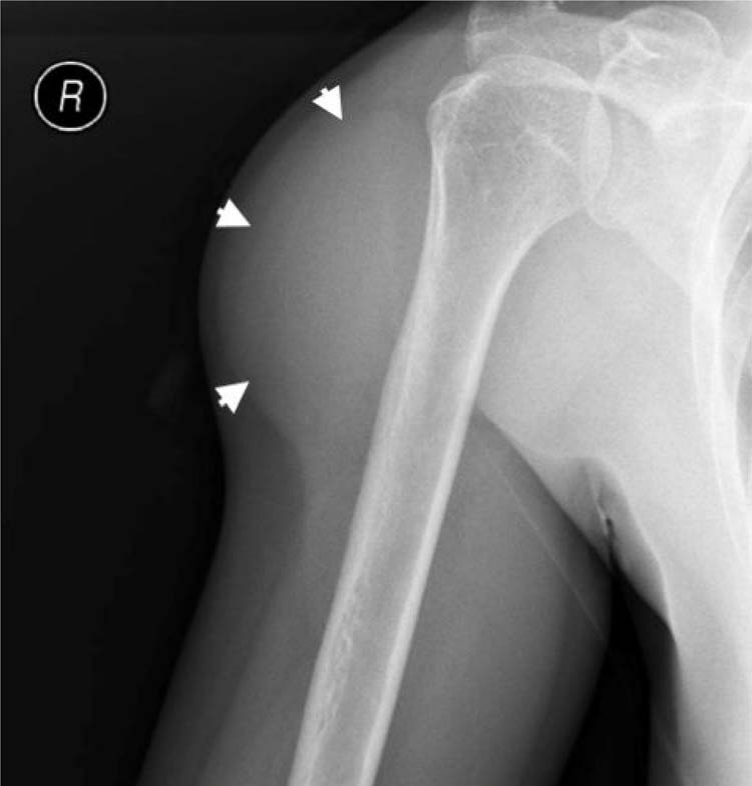
Externally rotated AP radiograph of the right shoulder and upper-arm. Short arrows indicate the tumoral mass.

**Figure 4:**
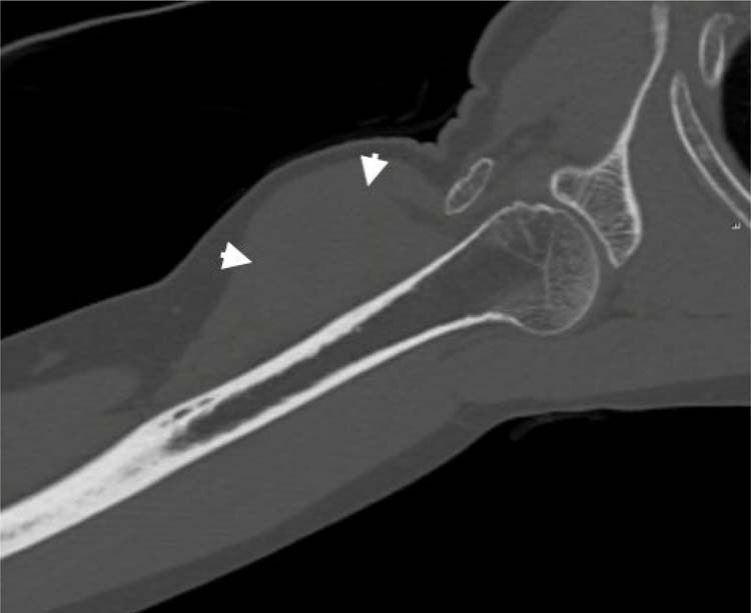
Coronal CT of the right arm. Short arrows indicate the tumoral mass.

Subsequent MRI showed a well-circumscribed ovoid mass beneath the deltoid muscle abutting the humeral bone without evidence of invasion of the bone or the adjacent neurovascular structures. The mass was heterogeneous isointense to muscle on T1 and heterogeneous hyper- and isointense to muscle on T2-fat-saturated images, embedded were multiple T1 and T2-fat-saturated hypointense foci, suggesting the possibility of internal calcifications or fibrous strands (Fig. 2A and C). The mass showed limited peripheral enhancement and septae, with a slow initial phase and plateau on dynamic contrast-enhanced images (Fig. 2B). No diffusion restriction was noted.

To exclude calcifications, a shoulder radiograph was carried out. The radiograph confirmed a soft tissue mass located around the proximal humeral bone with an estimated size of 9 × 5 cm, without radiographically visible calcifications (Fig. 3). A normal bone structure without periosteal reaction or cortical invasion was reported.

A successive non-contrast-enhanced computed tomography (CT) of the right upper arm confirmed the radiographic findings; a well-circumscribed isodense mass beneath the deltoid muscle without embedded calcifications or ossification (Fig. 4), the possibility of myositis ossificans was thereby excluded and a soft tissue tumor of fibroblastic origin became more likely.

### Pathophysiology

The excised tumor is a well-described nodular white mass of 94 grams, with measurements of 7.3 × 6.6 × 3.1 cm. The surface is white, with no tumor capsule found. Microscopic examination shows no infiltration of the muscular tissue. The tumor is hypocellular and consists of hyaline stroma and polygonal cells with small basophile core, neither clear mitosis nor obvious atypia. The architecture is vaguely nodular.

The specimen was referred to the pathophysiologic department of the University Hospital of Ghent. Protocol was completed as an encapsulated relatively hypocellular spindle cellular collagenous, partly myxoid spindle cellular mesenchymal tumoral proliferation of myofibroblast origin, confirming the diagnosis of a desmoplastic fibroblastoma.

### Differential diagnosis

Desmoid-type fibroma or sarcoma is the primary differential diagnosis to consider in these cases. Histology is subtly more vascularized and more cellular, with aggressive local infiltrative characteristics [5, 8]. Myoepithelioma is a benign spindle cell mass in a fibromyxoid stroma and can be found in extremities as well [9, 10]. MRI matches possible myositis ossificans, in possible post-traumatic setting. Myositis ossificans is a benign lesion in subcutaneous or muscular tissue with ossifying characteristics, usually self-limiting. Histopathology differs from time of diagnosis, but a wide array of imaging can help as diagnostic tool (CT scans, etc.) [11, 12].

### Treatment

The mass was excised under general anesthesia in outpatient clinical setting. Operative time was 40 minutes skin-to-skin. Surgical clips were left in the operative field, anticipating postoperative follow-up treatment. Hemostasis was controlled, and the wound was closed in layers over a drain. The drain was removed later that day. Our case was discussed multidisciplinary at the skin and soft-tissue malignancy team meeting. Dermatological and surgical follow-up was arranged, with a follow-up MRI 3 months postoperative.

## DISCUSSION

Our patient presented with a large mass when compared with other reported measurements, with average diameters of 2.0 to 5.5 cm in small case series [1, 13]. Sex remains an unclear predictor in the development of desmoplastic fibroblastoma, with a favor in prevalence for females [2, 5, 7, 13].

Junkins-Hopkins described the histology in 1998 as a hypocellular mass with spindled and stellated fibroblastic cells indiscriminately divided by fibrous and collagenous bundles [6]. Tumor growth is believed to be of neoplastic nature, since no signs of inflammation are present, in combination with atraumatic history [1]. This subsumes the idea that desmoplastic fibroblastoma is of reactive proliferatory origin.

Radiographs can identify soft-tissue nonspecific masses. When present, calcifications, osseous erosion of invasion can be detected [13]. CT imaging portrays the mass with a homogeneous structure, similar to skeletal musculature. The description of intramuscular desmoplastic fibroblastoma can show moderate heterogeneity with peripheral attenuation subtly greater than that of skeletal muscle [13].

MRI shows variable signaling depending on the sequence. Desmoplastic fibroblastoma shows weak to intermediate T1 signaling and a high T2 weighted signaling [5, 14, 15]. T1-weighted imaging shows a heterogenous consistency and signal intensity comparable to skeletal musculature [13]. The fat-suppressed imaging shows greater heterogeneity than the T1-weighted imaging, with a weaker signaling. Yamamoto et al. described a specific T1-weighted fat-suppressed peripheral rim post-contrast administration. They assume a difference in vascularization between the inner and utmost outer layer of the tumor [5]. With fat suppression, pathological processes tend to delineate vividly.

Immunohistochemical investigation confirms the diagnosis of desmoplastic fibroblastoma. Complete therapeutic approach equalizes a total resection [3, 5]. To this day, recurrence of the tumor after complete excision is not described. Follow-up imaging is generally not advised [7].

Desmoplastic firoblastoma is a rare soft benign tumor. The differential diagnosis includes other benign or low-grade fibroblastic/myofibroblastic lesions. MRI shows recognizable characteristics to help identification.
